# Inhibitory Effects of Constituents from the Aerial Parts of *Rosmarinus officinalis* L. on Triglyceride Accumulation

**DOI:** 10.3390/molecules22010110

**Published:** 2017-01-17

**Authors:** Jian Li, Tiwalade Adegoke Adelakun, Sijian Wang, Jingya Ruan, Shengcai Yang, Xiaoxia Li, Yi Zhang, Tao Wang

**Affiliations:** 1 Tianjin State Key Laboratory of Modern Chinese Medicine, 312 Anshanxi Road, Nankai District, Tianjin 300193, China; beyondwill@126.com (J.L.); tiwa_ade@yahoo.com (T.A.A.); Ruanjy19930919@163.com (J.R.); 15122473723@163.com (S.Y.); huifeidedouzi@yeah.net (X.L.); 2 Tianjin Key Laboratory of TCM Chemistry and Analysis, Institute of Traditional Chinese Medicine, Tianjin University of Traditional Chinese Medicine, 312 Anshanxi Road, Nankai District, Tianjin 300193, China; 15122587883@163.com

**Keywords:** *Rosmarinus officinalis*, flavonoids, terpenoids, phenolic acids, triglyceride accumulation inhibitory effects, HepG2 cells

## Abstract

Sixteen flavonoids (**1**–**16**) including two new ones, named officinoflavonosides A (**1**) and B (**2**) were obtained from the aerial parts of *Rosmarinus officinalis*. Among the known ones, **6**, **10**, and **13** were isolated from the rosmarinus genus for the first time. Their structures were elucidated by chemical and spectroscopic methods. Moreover, the effects on sodium oleate-induced triglyceride accumulation (TG) in HepG2 cells of the above-mentioned compounds and 16 other isolates (**17**–**32**) reported previously to have been obtained in the plant were analyzed. Results show that eight kinds of flavonoids (compounds **1**, **2**, **3**, **6**–**9** and **11**) and seven kinds of other known isolates (compounds **17**–**20**, **23**, **26** and **31**) possessed significant inhibitory effects on intracellular TG content in HepG2 cells. Among them, the activities of compounds **1** and **20** were comparable to that of orlistat, which suggested that these compounds in this plant might be involved in lipid metabolism.

## 1. Introduction

*Rosmarinus officinalis* L., is a perennial edible herb, belonging to Labiatae, commonly known as rosemary. *R. officinalis* extract has been reported to have antioxidant, anti-inflammatory, antidiabetic and anticancer properties [1]. In the course of our characterization studies on bioactive constituents from the aerial parts of *R. officinalis*, the isolation and structure elucidation of 16 terpenoids including normonoterpenoid, diterpenoid and triterpenoid glycosides [2], had been reported by us. Their structures were elucidated by chemical and spectroscopic methods. As a continuing study on the plant, we obtained 16 flavonoids including two new ones, named officinoflavonosides A (**1**) and B (**2**). In this paper, we describe the isolation and structure elucidation of these new flavanoids, along with the triglyceride (TG) accumulation inhibitory effects of the above-mentioned 45 compounds in HepG2 cells.

## 2. Results and Discussion

**Figure 1:**
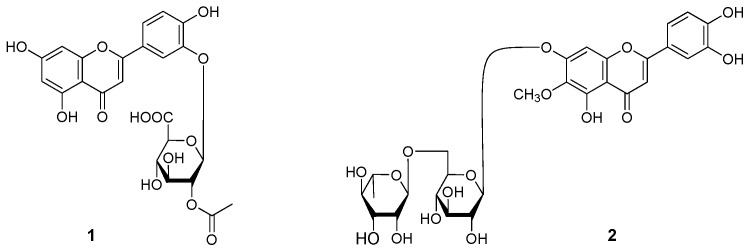
The new compounds **1** and **2** obtained from the aerial parts of *R. officinalis.*

**Figure 2:**
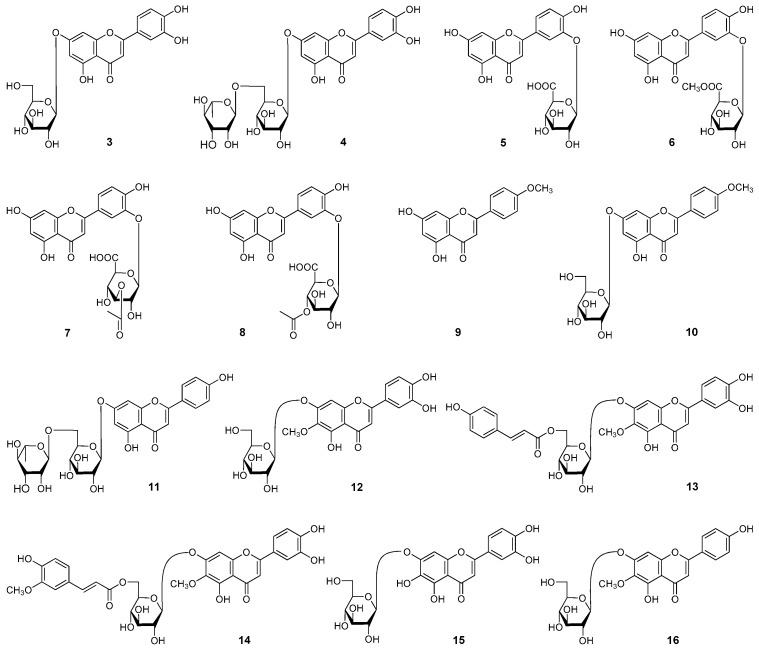
The known flavonoids (**3**–**16**) obtained from the aerial parts of *R. officinalis*.

During the course of our continuous studies on bioactive constituents from 95% EtOH eluate of D101 CC and CHCl_3_ layer [2,3] from *R. officinalis* aerial parts, 16 flavonoids, including two new compounds, named officinoflavonosides A (**1**) and B (**2**) (Figure 1), together with 14 known ones, luteolin-7-*O*-β-d-glucoside (**3**) [3], luteolin-7-*O*-β-d-lutinoside (**4**) [4], luteolin 3′-*O*-β-d-glucuronide (**5**) [5], 5,7,4′-trihydroxy-3′-*O*-β-d-glucuronic acid-6′′-methyl ester (**6**) [6], luteolin 3′-*O*-(3′′-*O*-acetyl)-β-d-glucuronide (**7**) [5], luteolin 3′-*O*-(4′′-*O*-acetyl)-β-d-glucuronide (**8**) [5], acacetin (**9**) [7], tiliadin (**10**) [8], apigenin-7-*O*-β-d-lutinoside (**11**) [4], nepitrin (**12**) [9], 6′′-*O*-(*E*)-*p*-coumaroylnepitrin (**13**), 6′′-*O*-(*E*)-feruloylnepitrin (**14**), 6-hydroxy luteolin-7-*O*-β-d-glucopyranoside (**15**) [10], and homoplantaginin (**16**) [11] (Figure 2) were further obtained. Among the known ones, **6**, **10**, and **13** were isolated from the rosmarinus genus 14 for the first time, and the NMR data of **13** and **14** in DMSO-*d*_6_ were reported for the first time.

**Figure 3:**
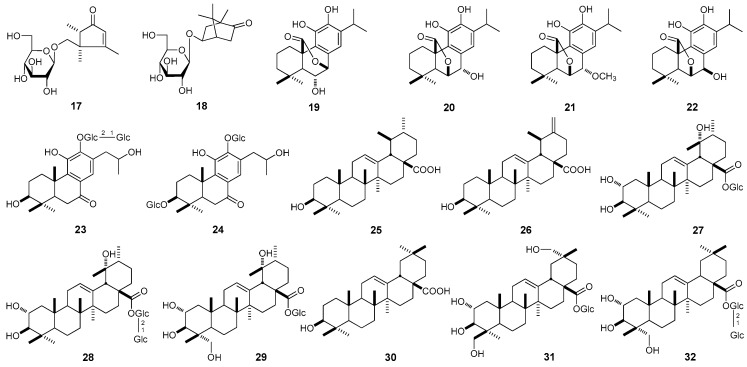
The known terpenoids (**17**–**32**) [2,3] obtained from the aerial parts of *R. officinalis* (Glc: β-d-glucopyranosyl).

This paper will elucidate the isolation and structure of new compounds, officinoflavonosides A (**1**) and B (**2**). Meanwhile, the effects of 16 flavonoids and 16 previously isolated terpenoids [2], officinoterpenoside D (1**7**), (1*S*,4*S*,5*S*)-5-exo-hydrocamphor 5-*O*-β-d-glucopyranoside (**18**), isorosmanol (**19**), rosmanol (**20**), 7-methoxyrosmanol (**21**), epirosmanol (**22**), officinoterpenosides A_1_ (**23**) and A_2_ (**24**), along with ursolic acid (**25**), micromeric acid (**26**), glucosyl tormentate (**27**), officinoterpenoside B (**28**), niga-ichigoside F_1_ (**29**), oleanolic acid (**30**), officinoterpenoside C (**31**), asteryunnanoside B (**32**) (Figure 3) on the reduction of TG content in HepG2 cells were determined.

**Figure 4:**
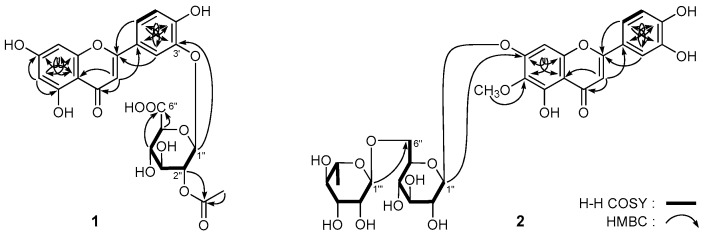
The main ^1^H-^1^H COSY and HMBC correlations of **1** and **2**.

*Officinoflavonoside A* (**1**) was isolated as an amorphous yellow powder with negative rotation {[α]${}_{D}^{25}$ –11.9° (*c* = 0.79, MeOH)}. Its molecular formula was determined to be C_23_H_20_O_1__3_ by negative-ion HRESI-TOF-MS (*m/z* 503.0838 [M − H]^−^, calcd for C_23_H_19_O_1__3_ 503.0831). Acid hydrolysis of **1** yielded d-glucuronic acid, which was identified by comparing the *R*_f_ value on TLC plate with the authentic sample. The ^1^H- and ^13^C-NMR (Table 1) spectra of **1**, which were assigned by various NMR experiments including ^1^H-^1^H COSY, HSQC, and HMBC spectra, indicated there was a luteolin part (δ 6.21 (1H, br. s, H-6), 6.51 (1H, br. s, H-8), 6.81 (1H, s, H-3), 7.01 (1H, d, *J* = 8.0 Hz, H-5'), 7.66 (1H, br. d, ca. *J* = 8 Hz, H-6′), 7.69 (1H, br. s, H-2′), 12.94 (1H, br. s, 5-OH)), a β-d-glucuronyl moiety (δ 5.39 (1H, d, *J* = 8.0 Hz, H-1′′)), together with an acetyl group (δ_H_ 2.07 (3H, s); δ_C_ 20.9 (CO*C*H_3_), 169.1 (*C*OCH_3_)) in the structure. A downfield shift of 0.3 ppm for the C-2′′, and upfield shift by 2.0 ppm both for the C-1′′ and 3′′ compared with those of luteolin 3′-*O*-β-d-glucuronide (**5**) [5], which indicated that the C-2′′ hydroxyl proton was substituted with an acetyl group. Finally, the linkages of β-d-glucuronyl moiety and acetyl group were clarified on the basis of HMBC experiment, which showed long-range correlations from δ_H_ 5.39 (H-1′′) to δ_C_ 144.9 (C-3′); δ_H_ 4.89 (1H, dd, *J* = 8.0, 8.0 Hz, H-2′′) to δ_C_ 169.1 (2-O*C*OCH_3_) (Figure 4). On the basis of the above-mentioned evidence, the structure of officinoflavonoside A was determined as luteolin 3′-*O*-(2′′-*O*-acetyl)-β-d-glucuronide (**1**).

*Officinoflavonoside B* (**2**) with negative optical rotation ([α]${}_{D}^{25}$ −47.4° in MeOH) was also isolated as an amorphous yellow powder. The molecular formula C_28_H_30_O_16_ of **2** was determined from negative-ion HRESI-TOF-MS (*m/z* 623.1595 [M − H]^−^, calcd for C_28_H_31_O_1__6_ 623.1618), too. The IR spectrum exhibited typical absorption bands of hydroxyl (3332 cm^−1^), ester carboxyl group (1658 cm^−1^), aromatic ring (1603, 1514, 1462 cm^−1^), and an *O*-glycosidic linkage (1067 cm^−1^). The UV data of **2** showed the characteristic maxima absorption of luteolin aglycon at 340 nm (4.17) and 267 nm (4.11). Acid hydrolysis of **2** with 1.0 M HCl liberated l-rhamnose and d-glucose, which were identified by HPLC analysis using an optical rotation detector [2]. The ^1^H- and ^13^C-NMR (Table 2) spectra of **2** indicated the presence of 6-methoxy luteolin (nepitrin) (δ 6.70 (1H, s, H-3), 6.89 (1H, s, H-8), 6.90 (1H, d, *J* = 8.0 Hz, H-5′), 7.40 (1H, dd, *J* = 2.0, 8.0 Hz, H-6′), 7.41 (1H, d, *J* = 2.0 Hz, H-2′)), and a β-d-glucopyranosyl (δ 5.13 (1H, d, *J* = 7.5 Hz, H-1′′)), together with an α-l-rhamnopyranosyl moiety (δ 1.05 (3H, d, *J* = 6.5 Hz, H-6′′′), 4.57 (1H, br. s, H-1′′′)). The connectivity of oligoglycoside moieties to the aglycon part was characterized by a HMBC experiment on **2**. Thus, the HMBC experiment of **2** showed long-range correlations between the following proton and carbon pairs (δ_H_ 5.13 (1′′-H) and δ_C_ 156.3 (C-7); δ_H_ 4.57 (H-1′′′) and δ_C_ 65.8 (C-6′′)) (Figure 4). Consequently, the structure of officinoflavonoside B was determined to be nepitrin 7-*O*-α-l-rhamnopyranosyl(1→6)-β-d-glucopyranoside (**2**).

**Figure 5:**
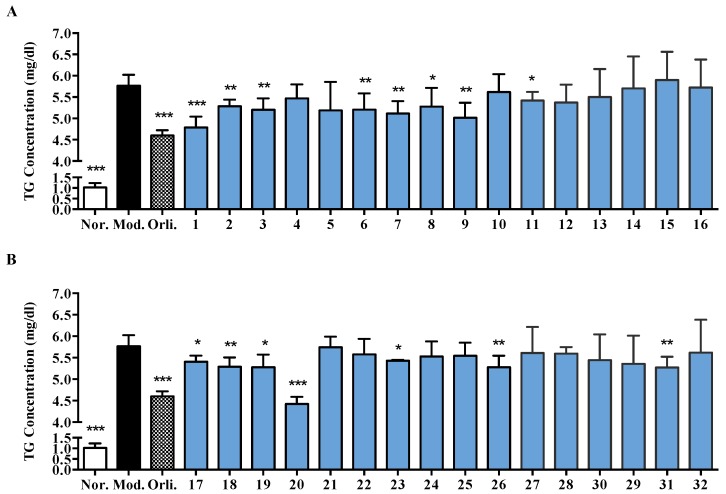
(**A**) Effects of 16 kinds of flavonoids (**1**–**16**) from *R. officinalis* on triglyceride (TG) accumulation in HepG2 cells; (**B**) Effects of 16 terpenoids (**17**–**32**) from *R. officinalis* on TG accumulation in HepG2 cells. Cells were incubated with 200 µmol/L SO for 48 h. Meanwhile, tested compounds (30 µmol/L) and positive-controlled orlistat (5 µmol/L, Orli.) were co-incubated to evaluate their inhibitory effects. Cells cultured in normal medium without sodium oleate (SO) were set as normal group (Nor.). The intracellular TG content in each well was examined using a TG assay kit. Each value represents the mean ± S.E.M., *n* = 5, *** *p* < 0.001, ** *p* < 0.01, * *p* < 0.05 vs. model group (Mod.).

TG accumulation of inhibitory effects of compounds isolated from aerial parts of *R. officinalis* were screened in sophisticated sodium oleate (SO)-induced HepG2 cells. The results showed that TG was accumulated as lipid droplets in the SO treated HepG2 cells monolayer and at least a 5-fold increase (*p* < 0.001) in TG content was observed from 51.14 ± 4.70 mg/dL (normal group) to 288.34 ± 5.72 mg/dL (model group). Compared to the SO group, orlistat significantly (*p* < 0.001) decreased this value to 230.06 ± 2.68 mg/dL by a 20.2% clearance ratio and eight of the 16 kinds of flavonoids tested showed similar inhibitory effects. Compounds **1**, **2**, **3**, **6**–**9** and **11** reduced the intracellular TG content by 17.0%, 8.4%, 9.8%, 9.7%, 11.3%, 8.5%, 13.0% and 6.0%, respectively, as given in Figure 5A. In addition, 16 terpenoids (**17**–**32**) were evaluated in the same method and seven kinds showed significantly inhibitory activities. The clearance ratios of **17**–**20**, **23**, **26** and **31** were 6.3%, 8.3%, 8.4%, 23.3%, 5.8%, 8.4% and 8.5%, respectively, as given in Figure 5B.

**Figure 6:**
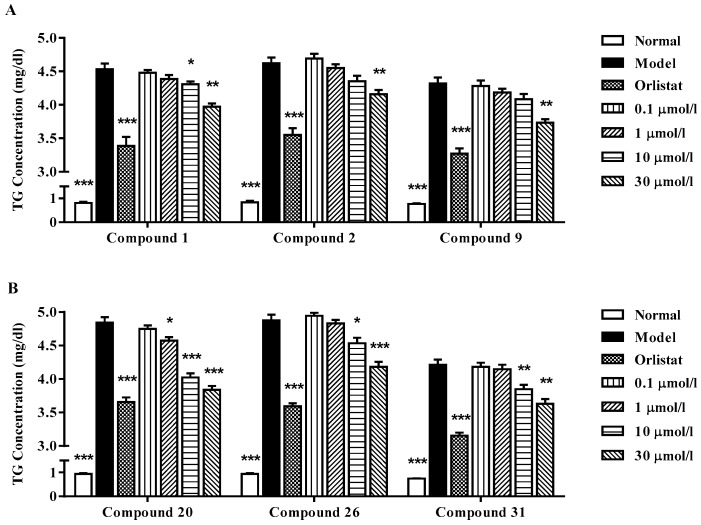
Dose dependency of TG-lowering effects of compounds **1**, **2**, **9**, **20**, **26** and **31** in HepG2 cells. HepG2 cells were treated with 0, 0.1, 1, 10 and 30 µmol/L of indicated compounds, respectively in the presence of SO for 48 h. Rate of TG lowering is given according to a formula: Rate of TG lowering (%) = ((TG concentration of untreated groups − TG concentration of treated groups)/TG concentration of untreated groups) × 100%. Each value represents the mean ± S.E.M., *n* = 5, *** *p* < 0.001 ** *p* < 0.01, * *p* < 0.05 vs. untreated controls. (**A**) effects of Compounds **1**, **2** and **9**; (**B**) effects of Compounds **20**, **26** and **31**.

**Figure 7:**
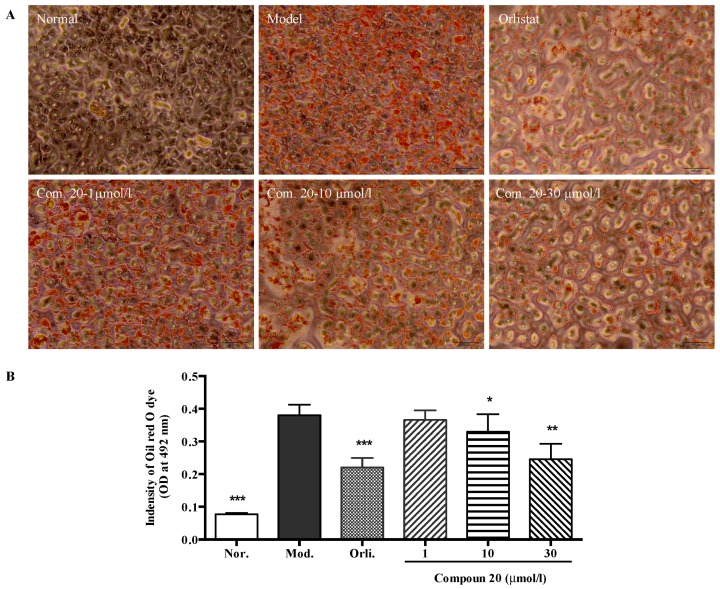
Effect of compound **20** from *R. officinalis* on TG accumulation in HepG2 cells. HepG2 cells were treated with indicated concentrations of orlistat (orlistat group, Orli.) or compound **20** in the absence (normal group, Nor.) or presence of SO for 48 h. Cells were stained with Oil Red O (**A**) and analyzed by colorimetric assay at 492 nm (**B**). Each value represents the mean ± S.E.M., *n* = 4, *** *p* < 0.001, ** *p* < 0.01, * *p* < 0.05 vs. model group (Mod.).

Studies of dose dependency of six selected compounds (compounds **1**, **2**, **9**, **20**, **26** and **31**), which comparatively showed the best TG-lowering activities, were then conducted. In this system, orlistat, a lipase inhibitor showed significant TG accumulation effects at concentration of 5 µmol/L. As shown in Figure 6, the results revealed that all the above isolates could inhibit the SO-induced intracellular TG accumulation in a dose dependent manner. Among them, compound **20** exhibited the strongest activity that it had demonstrated a clearance ratio of more than 5.6% from 1 µmol/L. Then, Oil red O staining further visually confirmed the results that a large amount of lipid droplets were seen in SO-treated HepG2 cells, indicating lipid accumulation, but the accumulation was inhibited by compound **20** in a dose dependent manner (shown in Figure 7). Although the colorimetric assay results showed that the concentration of 1 µmol/L exhibited no significant difference compared to the model group, the possible reasons for this might be the lower precision of this detection method or the activity-instability at this low concentration.

Although a limited number of compounds were tested for inhibitory effects on TG accumulation in HepG2 cells, we can summarize the structure-activities relationship as follows: in flavonoids, 5,7-dihydroxyl substitution showed strong activities (compounds **1**, **6**–**9**), and the activities would be reduced when 7-hydroxyl group was glucosylated (Compound **9** > **10**). Among the diterpenes, compound **20** showed strongest inhibitory effect on TG accumulation. Methylation or configuration changing at position 20 would quench the activity, which indicated that 20α-hydroxy is important for inhibitory activity.

## 3. Experimental Section

### 3.1. General

Optical rotations were determined on a Rudolph Autopol^®^ IV automatic polarimeter (l = 50 mm). IR spectra were recorded on a Varian 640-IR FT-IR spectrophotometer (Varian Australia Pty Ltd., Mulgrave, Australia). UV spectra were obtained on a Varian Cary 50 UV-Vis spectrophotometer (Varian, Inc., Hubbardsdon, MA, USA). NMR spectra were measured at a Bruker 500 MHz NMR spectrometer (Bruker BioSpin AG Industriestrasse 26 CH-8117, Fällanden, Switzerland) at 500 MHz for ^1^H- and 125 MHz for ^13^C-NMR, with TMS as an internal standard. Positive- and Negative-ion HRESI-TOF-MS were recorded on an Agilent Technologies 6520 Accurate-Mass Q-Tof LC/MS spectrometer (Agilent Corp., Santa Clara, CA, USA).

Column chromatographies (CC) were conducted on macroporous resin D101 (Haiguang Chemical Co., Ltd., Tianjin, China), Silica gel (74–149 μm, Qingdao Haiyang Chemical Co., Ltd., Qingdao, China), and ODS (50 μm, YMC Co., Ltd., Tokyo, Japan). Preparative HPLC (PHPLC) column, Cosmosil 5C_18_-MS-II (20 mm i.d. × 250 mm, Nakalai Tesque, Inc., Tokyo, Japan) was used to purify the constituents.

### 3.2. Plant Material

The dried aerial parts of *R*. *officinalis* were collected from Butarie, Rwanda, and identified by Dr. Li Tianxiang (Experiment Teaching Department, Tianjin University of Traditional Chinese Medicine). The voucher specimen was deposited at the Academy of Traditional Chinese Medicine of Tianjin University of TCM (No. 20110910).

### 3.3. Extraction and Isolation

The dried aerial parts of *R. officinalis* (2.5 kg) were dealt by using the same method as reported before [2,3]. As results, CHCl_3_ partition layer (269 g), H_2_O (47 g) and 95% EtOH (45 g) eluted fractions were obtained.

The EtOH fraction (36 g) was subjected to normal phase silica gel CC (CHCl_3_ → CHCl_3_–MeOH (100:3 → 100:5 → 100:7, *v*/*v*) → CHCl_3_–MeOH–H_2_O (10:3:1 → 7:3:1, *v*/*v*/*v*) → MeOH) to yield eleven fractions (Fr. 1–11). Fraction 8 (5480.0 mg) was subjected to PHPLC through gradient elution (MeOH–H_2_O (30:70 → 50:50 → 70:30 → 100:0, *v*/*v*)) to yield 22 fractions (Fr. 8-1–8-22). Fraction 8-15 (254.6 mg) was separated by PHPLC (CH_3_CN–H_2_O (21:79, *v*/*v*)) to give fractions 8-15-1 and 8-15-2. Fraction 8-15-1 (59.6 mg) was purified by PHPLC (MeOH–H_2_O (44:56, *v*/*v*)) and (CH_3_CN–H_2_O (19:81, *v*/*v*)) to yield homoplantaginin (**16**, 23.0 mg). Fraction 8-19 (228.0 mg) was purified by PHPLC (CH_3_CN–H_2_O (25:75, *v*/*v*)) to give 4′,5,7-trihydroxy-3′-*O*-β-d-glucuronic acid-6′′-methyl ester (**6**, 36.1 mg), tiliadin (**10**, 6.3 mg), 6′′-*O*-(*E*)-*p*-coumaroylnepitrin (**13**, 21.4 mg), and 6′′-*O*-(*E*)-feruloylnepitrin (**14**, 9.5 mg). Fraction 9 (10.0 g) was separated by ODS CC (MeOH–H_2_O (20:80 → 30:70 → 40:60 → 50:50 → 60:40 → 70:30 → 100:0, *v*/*v*)) to yield 14 fractions (Fr. 9-1–9-14). Fraction 9-10 (1510.0 mg) was purified by Sephadex LH-20 CC (CHCl_3_–MeOH (1:1, *v*/*v*)) to yield eight fractions (Fr. 9-10-1–9-10-8). Fraction 9-10-2 (512.2 mg) was subjected to PHPLC (MeOH–1% CH_3_COOH (45:55, *v*/*v*)] to obtain 14 fractions (Fr. 9-10-2-1–9-10-2-14). Fractions 9-10-2-5 (21.5 mg) and 9-10-2-6 (14.5 mg) were combined and subjected to PHPLC (CH_3_CN–1% CH_3_COOH (20:80, *v*/*v*)) to give homoplantaginin (**16**, 10.9 mg). Fraction 9-10-4 was separated by PHPLC (MeOH–1% CH_3_COOH (45:55, *v*/*v*)) to yield 13 fractions (Fr. 9-10-4-1–9-10-4-13). Fraction 9-10-4-12 (116.0 mg) was subjected to silica gel CC (CHCl_3_-MeOH-H_2_O (20:3:1, *v*/*v*/*v*) → MeOH) and PHPLC (CH_3_CN–1% CH_3_COOH (24:76, *v*/*v*)) to give luteolin 3′-*O*-β-d-glucuronide (**5**, 14.9 mg), luteolin 3′-*O*-(3′′-*O*-acetyl)-β-d-glucuronide (**7**, 14.5 mg), and luteolin 3′-*O*-(4′′-*O*-acetyl)-β-d-glucuronide (**8**, 9.3 mg). Fraction 10 (6.3 g) was isolated by PHPLC through gradient elution (MeOH–H_2_O (25:75 → 40:60 → 60:40→ 80:20 → 100:0, *v*/*v*)) to yield 35 fractions (Fr. 10-1–10-35). Fraction 10-24 (142.2 mg) was purified by Sephadex LH-20 CC (MeOH) and PHPLC (CH_3_CN–1% CH_3_COOH (16:84, *v*/*v*)) to yield 6-hydroxy luteolin-7-*O*-β-d-glucopyranoside (**15**, 9.0 mg). Fraction 10-25 (149.4 mg) was separated by PHPLC (CH_3_CN–1% CH_3_COOH (17:83, *v*/*v*)) to yield six fractions (Fr. 10-25-1–10-25-6). Fraction 10-25-5 (20.8 mg) was further purified by Sephadex LH-20 (MeOH–H_2_O (50:50, *v*/*v*)), and luteolin-7-*O*-β-d-lutinoside (**4**, 14.2 mg) was obtained. Fraction 10-26 (93.7 mg) was separated by PHPLC (CH_3_CN–1% CH_3_COOH (16:84, *v*/*v*)) to yield luteolin-7-*O*-β-d-glucoside (**3**, 23.6 mg). Fraction 10-27 (756.1 mg) was subjected to Sephadex LH-20 CC (MeOH) to yield nine fractions (Fr. 10-27-1–10-27-9). Fraction 10-27-5 (114.3 mg) was isolated by PHPLC (CH_3_CN–1% CH_3_COOH (17:83, *v*/*v*)) to give both officinoflavonoside B (**2**, 13.0 mg) and apigenin-7-*O*-β-d-lutinoside (**11**, 7.5 mg). Furthermore, fraction 10-27-7 (149.8 mg) was purified by PHPLC (CH_3_CN–1% CH_3_COOH (20:80, *v*/*v*)) to yield luteolin 3′-*O*-β-d-glucuronide (**5**, 28.6 mg) and nepitrin (**12**, 14.0 mg). Fraction 10-28 (473.3 mg) was also subjected to Sephadex LH-20 CC (MeOH) to yield seven fractions (Fr. 10-28-1–10-28-7). Fraction 10-28-6 (222.0 mg) was prepared by PHPLC (CH_3_CN–1% CH_3_COOH (20:80, *v*/*v*)) to give luteolin 3′-*O*-β-d-glucuronide (**5**, 13.5 mg). Furthermore, fraction 10-30 was isolated by Sephadex LH-20 CC (MeOH) and PHPLC (CH_3_CN–1% CH_3_COOH (25:75, *v*/*v*)) to yield officinoflavonoside A (**1**, 19.0 mg). Fraction 10-31 (198.5 mg) was separated by PHPLC (CH_3_CN–1% CH_3_COOH (26:74, *v*/*v*)) to give 4′,5,7-trihydroxy-3′-*O*-β-d-glucuronic acid-6′′-methyl ester (**6**, 24.0 mg).

The CHCl_3_ partition (200 g) of the rosemary extract was subjected to silica gel CC (CHCl_3_ → CHCl_3_–MeOH (100:1 → 100:3 → 100:5 →100:7, *v*/*v*) → CHCl_3_–MeOH–H_2_O (10:3:1 → 7:3:1, *v*/*v*/*v*) → MeOH) to yield 23 fractions (Fr. 1–23). Fraction 9 (56.3 g) was further subjected to silica gel CC (Pet. Ether (PE) → PE-EtOAc (20:1 → 15:1 → 10:1 → 5:1 → 3:1, *v*/*v*) → EtOAc) to yield 19 fractions (Fr. 9-1–9-19). Fraction 9-16 (5024.0 mg) was purified by PHPLC (MeOH–H_2_O (90:10, *v*/*v*)), and 13 fractions (Fr. 9-16-1–9-16-13) were given. Fraction 9-16-4 (629.8 mg) was subjected to PHPLC (MeOH–H_2_O (75:25, *v*/*v*)) to give acacetin (**9**, 2.6 mg).

*Officinoflavonoside A* (**1**): Yellow powder; [α]${}_{D}^{25}$ −11.9° (*c* = 0.79, MeOH); IR ν_max_ (KBr) cm^−1^: 3209, 2927, 2861, 1736, 1656, 1607, 1505, 1435, 1356, 1301, 1255, 1167, 1081, 1039; UV λ_max_ (MeOH) nm (log *ε*): 335 (4.20), 265 (4.15). ^1^H-NMR (500 MHz, DMSO-*d*_6_) and ^13^C-NMR (125 MHz, DMSO-*d*_6_) spectroscopic data, see Table 1; HRESI-TOF-MS: Negative-ion mode *m/z* 503.0838 [M – H]^−^ (calcd for C_23_H_19_O_13_ 503.0831).

*Officinoflavonoside B* (**2**): Yellow powder; [α]${}_{D}^{25}$ −47.4° (*c* = 0.59, MeOH); IR ν_max_ (KBr) cm^−1^: 3332, 2923, 1658, 1603, 1570, 1514, 1462, 1355, 1276, 1192, 1067, 1014; UV λ_max_ (MeOH) nm (log *ε*): 340 (4.17), 267 (4.11). ^1^H-NMR (500 MHz, DMSO-*d*_6_) and ^13^C-NMR (125 MHz, DMSO-*d*_6_) spectroscopic data, see Table 2; HRESI-TOF-MS: Negative-ion mode *m/z* 6231595 [M – H]^−^ (calcd for C_28_H_31_O_16_ 623.1618).

*6*′′*-O-(E)-p-Coumaroylnepitrin* (**13**): Yellow powder; The NMR data of **13** in DMSO-*d*_6_ are reported for the first time. ^1^H-NMR (DMSO-*d*_6_, 500 MHz): δ 6.70 (1H, s, H-3), 6.96 (1H, s, H-8), 7.45 (2H, m, H-2′ and 6′), 6.89 (1H, d, *J* = 9.0 Hz, H-5′), 5.23 (1H, d, *J* = 7.0 Hz, H-1′′), 3.41 (1H, dd, *J* = 7.0, 9.0 Hz, H-2′′), 3.39 (1H, dd, *J* = 9.0, 9.0 Hz, H-3′′), 3.30 (1H, dd, *J* = 9.0, 9.0 Hz, H-4′′), 3.86 (1H, m, H-5′′), (4.25 (1H, dd, *J* = 7.0, 12.0 Hz), 4.43 (1H, br. d, ca. *J* = 12 Hz), H_2_-6′′), 7.27 (2H, d, *J* = 8.5 Hz, H-2′′′,6′′′), 6.60 (2H, d, *J* = 8.5 Hz, H-3′′′,5′′′), 7.46 (1H, d, *J* = 16.0 Hz, H-7′′′), 6.29 (1H, d, *J* = 16.0 Hz, H-8′′′), 3.77 (3H, s, 6-OC*H*_3_), 13.03 (1H, br. s, 5-OH). ^13^C-NMR (DMSO-*d*_6_, 125 MHz): δ 164.5 (C-2), 102.3 (C-3), 182.0 (C-4), 152.5 (C-5), 132.5 (C-6), 156.1 (C-7), 94.0 (C-8), 152.0 (C-9), 105.7 (C-10), 120.8 (C-1′), 113.1 (C-2′), 145.9 (C-3′), 150.5 (C-4′), 115.9 (C-5′), 119.0 (C-6′), 99.8 (C-1′′), 72.9 (C-2′′), 76.3 (C-3′′), 69.9 (C-4′′), 73.7 (C-5′′), 63.4 (C-6′′), 124.6 (C-1′′′), 115.5 (C-2′′′,6′′′), 129.9 (C-3′′′,5′′′), 159.7 (C-4′′′), 144.9 (C-7′′′), 113.4 (C-8′′′), 166.4 (C-9′′′), 60.2 (6-O*C*H_3_). Negative-ion mode *m/z* 623.1413 [M − H]^−^ (calcd for C_31_H_27_O_14_ 623.1406).

*6*′′*-O-(E)-Feruloylnepitrin* (**14**): Yellow powder; The NMR data of **14** in DMSO-*d*_6_ are reported for the first time. ^1^H-NMR (DMSO-*d*_6_, 500 MHz): δ 6.63 (1H, s, H-3), 6.95 (1H, s, H-8), 7.39 (1H, br. s, H-2′), 6.83 (1H, d, *J* = 8.0 Hz, H-5′), 7.39 (1H, br. d, ca. *J* = 8 Hz, H-6′), 5.23 (1H, d, *J* = 7.0 Hz, H-1′′), 3.40 (1H, dd, *J* = 7.0, 9.0 Hz, H-2′′), 3.38 (1H, dd, *J* = 9.0, 9.0 Hz, H-3′′), 3.32 (1H, dd, *J* = 9.0, 9.0 Hz, H-4′′), 3.83 (1H, m, H-5′′), (4.24 (1H, dd, *J* = 6.5, 12.0 Hz), 4.44 (1H, br. d, ca. *J* = 12 Hz), H_2_-6′′), 7.13 (1H, br. s, H-2′′′), 6.60 (1H, d, *J* = 8.0 Hz, H-5′′′), 6.83 (1H, br. d, ca. *J* = 8 Hz, H-6′′′), 7.45 (1H, d, *J* = 16.0 Hz, H-7′′′), 6.37 (1H, d, *J* = 16.0 Hz, H-8′′′), 3.76 (3H, s, 6-OC*H*_3_), 13.07 (1H, br. s, 5-OH), 3.71 (3H, s, 3′′′-OC*H*_3_). ^13^C-NMR (DMSO-*d*_6_, 125 MHz): 164.6 (C-2), 102.1 (C-3), 182.0 (C-4), 152.4 (C-5), 132.4 (C-6), 156.1 (C-7), 94.0 (C-8), 152.0 (C-9), 105.7 (C-10), 120.6 (C-1′), 113.0 (C-2′), 146.0 (C-3′), 150.5 (C-4′), 115.8 (C-5), 119.1 (C-6′), 99.8 (C-1′′), 73.0 (C-2′′), 76.3 (C-3′′), 69.7 (C-4′′), 73.7 (C-5′′), 63.1 (C-6′′), 125.2 (C-1′′′), 111.0 (C-2′′′), 147.7 (C-3′′′), 149.3 (C-4′′′), 115.3 (C-5′′′), 122.7 (C-6′′′), 145.2 (C-7′′′), 113.8 (C-8′′′), 166.4 (C-9′′′), 60.2 (6-O*C*H_3_), 55.4 (3′′′-O*C*H_3_). Negative-ion mode *m/z* 653.1514 [M − H]^−^ (calcd for C_32_H_29_O_15_ 653.1512).

Acid Hydrolysis of **1** and **2**. A solution of officinoflavonoside A (**1**, 2.0 mg) was hydrolyzed with 10% H_2_SO_4_ in 50% EtOH (2 mL) at 100 °C for 3 h. The reaction mixture was neutralized with ion exchange resin Amberlite IRA-400 (OH^−^ form) and evaporated to dryness. The sugar was found to be glucuronide by comparison of the *R*_f_ value (*R*_f_ = 0.21) on TLC plate (silica gel, CHCl_3_–MeOH–H_2_O (6:4:1, *v*/*v*/*v*, lower layer)) with the authentic d-glucuronide.

Meanwhile, a solution of officinoflavonoside B (**2**, 2.0 mg) in 1 M HCl (1 mL) was heated under reflux for 3 h, respectively. The reaction mixture was dealt with the similar methods as those used to officinoterpenosides A–D [2]. Identification of l-rhamnose (i) and d-glucose (ii) from **2** presented in the aqueous was carried out by comparison of its retention time and optical rotation with that of authentic samples, *t*_R_: (i) 9.1 min (negative, l-rhamnose), and (ii) 17.6 min (positive, d-glucose).

### 3.4. Evaluation of Effects on Sodium oleate-induced TG Accumulation in HepG2 Cells

*Materials.* A human hepatoma HepG2 cell line was obtained from Cell Resource Center of Institute of Basic Medical Sciences, Chinese Academy of Medical Sciences & Peking Union Medical College (Beijing, China). Dulbecco’s modified Eagle’s medium (DMEM), penicillin and streptomycin were purchased from Thermo SCIENTIFIC (Waltham, MA, USA). Fetal Bovine Serum (FBS) was purchased from PAN-Biotech GmbH (Bavaria, Germany). TG assay kits were purchased from Wako Pure Chemical Industries, Ltd. (Osaka, Japan). Sodium oleate and orlistat were obtained from Sigma-Aldrich Corporation (St. Louis, MO, USA).

*Cell culture*. HepG2 cells were grown in DMEM supplemented with 10% FBS and 1% antibiotics (100 unit/mL penicillin and 100 mg/mL streptomycin). Cells were maintained in an atmosphere of 95% air and 5% CO_2_ at 37 °C in subconfluent condition.

*Sodium oleate-induced TG accumulation*. TG accumulation inhibitory effects were screened as previous report [12]. Briefly, Cells were seeded at a density of 100,000 cells/mL on Corning 48-multiwell plates. After 24 h in culture, medium was exchanged for phenol red free-DMEM with or without Sodium oleate (200 µmol/L) in the presence or absence of the obtained isolates (30 µmol/L) from *R. officinalis* for another 48 h. Meanwhile, an anti-obesity drug, orlistat (5 µmol/L), was used as a positive control. Cells were then rinsed with phosphate buffered saline twice and 200 µL deionized water was added per well to get cell lysate by ultrasonication. Intracellular TG contents in the lysates were finally evaluated using a GPO-POD method as the kit protocol provided. Briefly, the method is based on the enzymatic hydrolysis of triglyceride to glycerol and free fatty acids by lipoprotein lipase. The glycerol is phosphorylated by adenosine triphosphate in the presence of glycerolkinase to form glycerol-3-phosphate and adenosine diphosphate. glycerol-3-phosphate is oxidized by glycerophosphate oxidase (GPO) to form dihydroxyacetone phosphate and hydrogen peroxide. Purple quinoneimine complex is produced by the peroxidase (POD) catalyzed coupling of and 4AAP and ESPAS with hydrogen peroxide. The final absorbance was monitored at 492 nm using a microplate reader (Molecular Devices, Sunnyvale, CA, USA). Concentration setting of test sample and orlistat were established by pre-test results. Under the concentration, there were no treatment-related changes in cell viability (Data not shown).

*Oil red O staining* After a 48 h-treatment in 24-multiwell plates, cells were washed with phosphate-buffered saline (PBS) and fixed in 10% paraformaldehyde for 30 min at room temperature, followed by washing twice with deionized water and incubated in Oil Red O working solution (water at a 3:2 ratio of stock solution, which prepared by 0.5% Oil Red O dye in isopropanol) for 1 h. The unbound dye was removed by washing with 75% ethyl alcohol for once and images were immediately captured under an inverted phase contrast microscope (Leica, Wetzlar, Germany). For quantitative analysis, stained cells were then dissolved in isopropanol and absorbance was measured at 492 nm.

### 3.5. Statistical Analysis

Statistical analysis was performed using one-way ANOVA with the Tukey’s test for multiple comparisons. All data were expressed as mean ± S.E.M. and values of *p* < 0.05 were considered significant.

## 4. Conclusions

Taken together, 16 flavonoids (**1**–**16**) including two new ones, officinoflavonosides A (**1**) and B (**2**), were obtained from the aerial parts of *R. officinalis*. Among the known ones, **6**, **10**, and **13** were isolated from the rosmarinus genus for the first time, and the NMR data of **13** and **14** in DMSO-*d*_6_ were reported for the first time. Their structures were elucidated by chemical and spectroscopic methods and their effects on TG accumulation were analyzed in HepG2 cells. The results showed that six kinds of flavonoids (compounds **3**, **6**, **7**, **8**, **9** and **11**) together with the two novel ones (compounds **1** and **2**) and seven kinds of other known isolates (compounds **17**, **18**, **19**, **20**, **23**, **26** and **31**) possessed significant inhibitory activities on TG accumulation in vitro. Among them, the activities of compounds **1** and **20** were comparable to that of orlistat, which suggested that these compounds contained in the *R*. *officinalis* might be involved in the lipid metabolism.

## Figures and Tables

**Table 1:** ^1^H- and ^13^C-NMR data for **1** in DMSO-*d*_6_.

No.	δ_C_	δ_H_ (*J* in Hz)	No.	δ_C_	δ_H_ (*J* in Hz)
2	163.2	-	4′	151.7	-
3	103.1	6.81 (s)	5′	116.8	7.01 (d, 8.0)
4	181.7	-	6′	122.4	7.66 (br. d, ca. 8)
5	161.4	-	1′′	98.8	5.39 (d, 8.0)
6	98.8	6.21 (br. s)	2′′	73.2	4.89 (dd, 8.0, 8.0)
7	164.2	-	3′′	73.4	3.57 (dd, 8.0, 8.0)
8	94.0	6.51 (br. s)	4′′	71.5	3.55 (dd, 8.0, 8.0)
9	157.2	-	5′′	75.2	4.01 (d, 8.0 Hz)
10	103.6	-	6′′	170.3	-
1′	121.3	-	2′′-*C*OCH_3_	169.1	-
2′	115.7	7.69 (br. s)	2′′-CO*C*H_3_	20.9	2.07 (s)
3′	144.9	-	5-OH	-	12.94 (br. s)

**Table 2:** ^1^H- and ^13^C-NMR data for **2** in DMSO-*d*_6_.

No.	δ_C_	δ_H_ (*J* in Hz)	No.	δ_C_	δ_H_ (*J* in Hz)
2	164.8	-	1′′	100.3	5.13 (d, 7.5)
3	102.4	6.70 (s)	2′′	73.1	3.32 (m, overlapped)
4	182.0	-	3′′	76.4	3.32 (m, overlapped)
5	152.7	-	4′′	69.4	3.21 (dd, 9.0, 9.0)
6	132.7	-	5′′	75.5	3.61 (m)
7	156.3	-	6′′	65.8	3.51 (dd, 4.5, 12.0)
8	94.2	6.89 (s)			3.85 (br. d, ca. 12)
9	152.1	-	1′′′	100.3	4.57 (br. s)
10	105.8	-	2′′′	70.3	3.64 (br. d, ca. 3)
1′	120.7	-	3′′′	70.7	3.46 (dd, 3.0, 9.0)
2′	113.2	7.41 (d, 2.0)	4′′′	71.9	3.15 (dd, 9.0, 9.0)
3′	146.2	-	5′′′	68.2	3.42 (m)
4′	150.8	-	6′′′	17.7	1.05 (d, 6.5)
5′	115.9	6.90 (d, 8.0)	6-OCH_3_	60.2	3.77 (s)
6′	119.0	7.40 (dd, 2.0, 8.0)	5-OH	-	13.06 (br. s)
